# The Ariadne principles: how to handle multimorbidity in primary care consultations

**DOI:** 10.1186/s12916-014-0223-1

**Published:** 2014-12-08

**Authors:** Christiane Muth, Marjan van den Akker, Jeanet W Blom, Christian D Mallen, Justine Rochon, François G Schellevis, Annette Becker, Martin Beyer, Jochen Gensichen, Hanna Kirchner, Rafael Perera, Alexandra Prados-Torres, Martin Scherer, Ulrich Thiem, Hendrik van den Bussche, Paul P Glasziou

**Affiliations:** Institute of General Practice, Johann Wolfgang Goethe University, Theodor-Stern-Kai 7, D-60590 Frankfurt, Germany; School CAPHRI, Department of Family Medicine, Maastricht University, P.O. Box 616, 6200 MD Maastricht, The Netherlands; Department of General Practice, Katholieke Universiteit Leuven, Kapucijnenvoer 33, blok J, 3000 Leuven, Belgium; Department of Public Health and Primary Care, Leiden University Medical Center, Postbus 9600, 2300 RC Leiden, The Netherlands; Research Institute for Primary Care and Health Sciences, Keele University, Staffordshire, ST5 5BG UK; Institute of Medical Biometry and Informatics (IMBI), University of Heidelberg, Im Neuenheimer Feld 305, D‐69120 Heidelberg, Germany; Netherlands Institute for Health Services Research (NIVEL), Postbus 1568, 3500BN Utrecht, The Netherlands; Department of General Practice and Elderly care medicine/EMGO Institute for Health and Care Research, VU University Medical Center, Van der Boechorststraat 7, 1081 BT Amsterdam, The Netherlands; Department of Family Medicine, Preventive and Rehabilitative Medicine, Philipps University of Marburg, Karl-von-Frisch-Str. 4, D-35043 Marburg, Germany; Department of General Practice and Family Medicine, University Hospital, Friedrich Schiller University, Bachstraße 18, D-07740 Jena, Germany; Department of Primary Care Health Sciences, University of Oxford, 23-38 Hythe Bridge Street, Oxford, OX1 2ET UK; EpiChron Research Group on Chronic Diseases, Aragon Health Sciences Institute, IIS Aragón, Paseo Isabel La Católica 1-3, 50009 Zaragoza, Spain; Department of Primary Medical Care, University Medical Centre Hamburg-Eppendorf, Martinistraße 52, 20246 Hamburg, Germany; Department of Medical Informatics, Biometry and Epidemiology, Ruhr University of Bochum, Overbergstr. 17, 44801 Bochum, Germany; Department of Geriatrics, Marienhospital Herne, Clinical Centre of the Ruhr University, Widumer Str. 8, 44627 Herne, Germany; The Centre for Research in Evidence-Based Practice (CREBP), Bond University, Gold Coast, Robina QLD 4226 Australia

**Keywords:** Comorbidity, Decision making, General practice, Goal-oriented care, Multimorbidity, Patient-centered care, Patient care planning, Patient preference, Primary care

## Abstract

Multimorbidity is a health issue mostly dealt with in primary care practice. As a result of their generalist and patient-centered approach, long-lasting relationships with patients, and responsibility for continuity and coordination of care, family physicians are particularly well placed to manage patients with multimorbidity. However, conflicts arising from the application of multiple disease oriented guidelines and the burden of diseases and treatments often make consultations challenging. To provide orientation in decision making in multimorbidity during primary care consultations, we developed guiding principles and named them after the Greek mythological figure Ariadne. For this purpose, we convened a two-day expert workshop accompanied by an international symposium in October 2012 in Frankfurt, Germany. Against the background of the current state of knowledge presented and discussed at the symposium, 19 experts from North America, Europe, and Australia identified the key issues of concern in the management of multimorbidity in primary care in panel and small group sessions and agreed upon making use of formal and informal consensus methods. The proposed preliminary principles were refined during a multistage feedback process and discussed using a case example. The sharing of realistic treatment goals by physicians and patients is at the core of the Ariadne principles. These result from i) a thorough interaction assessment of the patient’s conditions, treatments, constitution, and context; ii) the prioritization of health problems that take into account the patient’s preferences – his or her most and least desired outcomes; and iii) individualized management realizes the best options of care in diagnostics, treatment, and prevention to achieve the goals. Goal attainment is followed-up in accordance with a re-assessment in planned visits. The occurrence of new or changed conditions, such as an increase in severity, or a changed context may trigger the (re-)start of the process. Further work is needed on the implementation of the formulated principles, but they were recognized and appreciated as important by family physicians and primary care researchers.

Please see related article: http://www.biomedcentral.com/1741-7015/12/222.

**Electronic supplementary material:**

The online version of this article (doi:10.1186/s12916-014-0223-1) contains supplementary material, which is available to authorized users.

## Multimorbidity in primary care consultation

Multimorbidity, the co-occurrence of multiple chronic conditions in an individual, is a health issue mostly dealt with in family practice [1-4]. As a result of their generalist and patient-centered approach, long-lasting relationships with patients, and responsibility for continuity and coordination of care, family physicians are particularly well placed to manage patients with multimorbidity [5]. However, physicians can feel overwhelmed by multimorbidity, specifically the challenges of identifying the inter-dependence between current and continuing problems, managing multiple changing conditions, and the interplay between psychosocial issues (including motivation and empowerment) and therapeutic and prognostic aspects [6-8].

Patients with chronic diseases often suffer from the cumulative burden of their treatments as well as any primary and secondary prevention, prompting a call for a minimally disruptive medicine approach [9,10]. The potential mismatch between patients’ and doctors’ preferences and priorities [11] and conflicts between single-disease guideline recommendations make each consultation with a patient who has multimorbidity more demanding than those with patients with a single disease [6,8,12-14]. Although family physicians have devised ways to manage patients with multimorbidity, it is rarely actively considered in medical decision making [15].

To unpick the complexity of the management of multimorbidity, we can focus on the decisions made by patients and doctors during consultation. One model of the decisions required in a comprehensive model of primary care consultations was developed by Stott and Davis in the 1970s, and is still taught and applied [16]. Given that current disease-oriented guidelines do not account for the interactions between the different diseases [12,17], a framework for a different consultation model was recently proposed for geriatric patients with multimorbidity [18]. This approach has yet to be considered for patients in primary care consultations that require a longitudinal and comprehensive approach [5,19,20]. Therefore, we set out to develop a tool to support decision-making during consultations in primary care that involve patients with multimorbidity.

### Process of development

Rather than use a formal consensus approach, we designed a process aimed at fostering the re-conceptualization of medical decision making in patients with multimorbidity in primary care. Our description of methods aims to raise the transparency of this informal, multi-stage process. For the initial development process we convened a two-day expert workshop, which was preceded by an international symposium in October 2012 in Frankfurt, Germany. The first phase consisted of a one-day symposium to provide an initial exchange of ideas between speakers, and a wider audience. At the symposium, the current state of knowledge on the prevalence and patterns of multimorbidity, the complex problems of multimorbidity management and its associated polypharmacy, the inappropriateness of disease-oriented clinical practice guidelines, and the challenges involved in applying evidence-based medicine to individual patients with multimorbidity were summarized within 12 presentations and discussed with a broad international audience (for the detailed program, see: [21]). Following the symposium, nineteen workshop participants from six countries (Australia, Canada, Germany, the Netherlands, Spain, and the UK) used panel and small group discussions to identify the key issues of concern relating to medical decision making in patients with multimorbidity in primary care. The workshop participants represented the fields of primary care, public health, and geriatrics – with a focus on epidemiology, evidence-based medicine, and methodology. The discussion was facilitated by an independent moderator who used both informal and formal techniques (e.g., nominal group processes). Over the following eight months, we drafted the principles. In June and July 2013, we circulated the proposed preliminary results to practicing family physicians and other experts in six countries and asked for a structured feedback on appropriateness, feasibility, and comprehensiveness in the form of ratings and free text comments. Taking into consideration the results of the written external feedback of 24 respondents (Additional file 1), we refined the principles. Written informed consent was obtained from the patient/participant for publication of their individual details in this manuscript. We then discussed the refined principles with other family physicians and independent experts in four group discussions that took place at Gold Coast and Newcastle (Australia) and Frankfurt/Main and Bad Schwalbach (Germany) using the case example. The key issues that came to light during the group discussions were passed on to all authors and agreement was sought on necessary changes to the manuscript. This paper reports on the key principles that emerged from this 14-month iterative process to provide guidance on multimorbidity management for family physicians in their context-specific clinical decision making.

### Tasks of primary care consultation

Stott and Davis described a widely used framework aimed at helping family physicians to broaden the consultation beyond the presenting complaint with the four following elements: i) management of the presenting problems, ii) management of continuing problems, iii) modification of help-seeking behaviors, and iv) opportunistic health promotion [16].

#### Management of the presenting problem(s)

**Figure 1:**
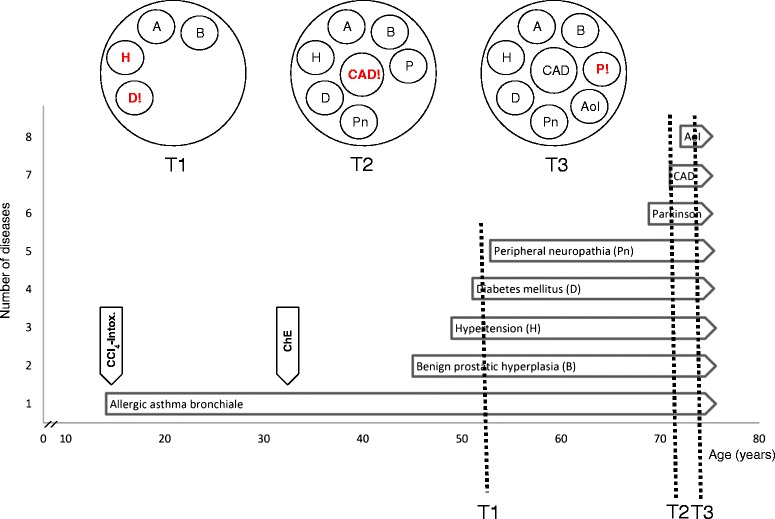
**Life-time medical history of Mr. P.** T1 to T3: Visits with patient at three different times (see text). AoI, Aortic insufficiency; B, Benign prostatic hyperplasia; CAD, Coronary artery disease; CCl4 Intox., Accidental intoxication with carbon tetrachloride; ChE, Cholecystectomy; D, Diabetes mellitus; H, Hypertension; P, Parkinson disease; Pn, Peripheral neuropathia. **Mr. P** is a 77-year-old, married and highly educated man living at home with his wife. The course of his medical history is depicted in Figure 1. We selected three periods of Mr. P’s history (T1, T2, and T3): **At T1**, Mr. P is 52 years old and the main focus of his medical care lies on his diabetes and hypertension. He measures his blood glucose level and blood pressure on a daily basis. He takes oral hypoglycemics and antihypertensives, and follows dietary restrictions. For asthma control he uses inhalers. His benign prostatic hyperplasia is only mildly symptomatic. **At T2**, Mr. P is a 71-year-old pensioner who has been admitted to hospital with angina pectoris. A two-vessel coronary artery disease (CAD) is diagnosed, and Mr. P is discharged after a percutaneous coronary intervention that included stent implantation (Stent-PCI) at one vessel. Ten months later, he is re-admitted with angina pectoris. Another Stent-PCI is conducted and a beta-blocker is prescribed due to the CAD progression. Since T1, a primary Parkinson syndrome and a peripheral neuropathy have been newly diagnosed. The number of prescriptions has risen from 5 oral drugs to 11. **At T3**, Mr. P is 75 years old. He presents with a cough, problems swallowing and hypersalivation, increased stiffness, severe back pain, fluctuating blood pressure, and low mood. He needs help with most activities of daily living and finds it increasingly difficult to follow his treatment plan (encompassing 14 oral drugs, and two inhalers with seven times daily dosing). At a special care unit for Parkinson’s, his medication has been changed completely. The administration of amantadine resulted in urinary retention, requiring the insertion of a transitory indwelling urine catheter. After drug withdrawal, the catheter could be removed. He has physical therapy and is discharged with reduced symptoms of Parkinson’s (reduced stiffness, coughing, and back pain; no problems with hypersalivation and swallowing), increased functionality and mood, a treatment plan consisting of 12 drugs, six times a day, and no ongoing problems of urinary retention or fluctuations in blood pressure. To date, he has no cognitive deficits and conducts all (instrumental) activities of daily living with reduced speed but without external support. He practices physical exercise daily and is well integrated socially.

Dealing with newly presented problems may be complicated by the presence of multimorbidity, as the presenting problem might arise from one of the patient’s existing diseases or from treatments of those diseases. In our case example (Figure 1), Mr. P’s presenting problem of symptomatic fluctuating blood pressure might have been caused by his Parkinson’s disease or was perhaps due to inappropriate antihypertensive medication; moreover, his cough may have indicated a deterioration in his Parkinson’s disease, or a potential worsening of his asthma due to treatment with beta-blockers.

#### Management of continuing problems

Patients with multimorbidity will also require attention to ongoing management of their other problems, including a check on progress, adherence to treatment, and any mention of secondary prevention. This will compete for time during the consultation, and require careful prioritization. In our case example, Mr. P’s may also require attention to his Parkinson’s disease or asthma.

#### Modification of help-seeking behavior

Every doctor-patient encounter should conclude by checking and negotiating the patient’s needs and expectations with regard to future consultations, including routine visits and ‘safety netting’. It is important to avoid an excessively high treatment burden of patients that interferes with their daily life and results in adherence problems with treatments and appointments, both for presenting problems and ongoing diseases. For example, Mr. P is highly motivated and adheres to his treatment plan. However, at T3 he was unable to cope with either the treatment plan or ambulatory appointments.

#### Opportunistic health promotion

Preventive activities should include appropriate age- and sex-specific prevention, but the presence of multimorbidity may overload patients and physicians. For instance, for Mr. P, at least seven primary prevention measures are recommended including a screening for colon cancer, osteoporosis and kidney disease, and visual and hearing impairment, as well as a fall assessment and a comprehensive eye examination [22].

### The Ariadne principles of counseling for patients with multimorbidity

In Greek mythology, Ariadne helped Theseus to find his way out of the Minotaur’s labyrinth by giving him a ball of thread – a picture that fits in well with multimorbidity research [23]. Our Ariadne principles can be viewed as the thread that helps the physician to find his/her way within the labyrinth of multiple primary care consultations and (patient) contacts to other health care professionals. Often, it is not feasible (nor desirable) to work-up all elements of a consultation within one appointment. Our principles are not limited to one consultation, but are expected to be applied continuously while emphasizing certain aspects in each consultation.

**Figure 2:**
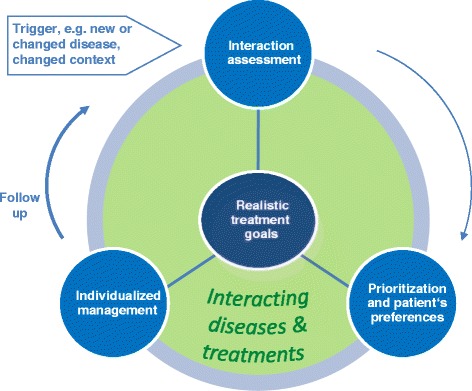
**Ariadne principles.**

Figure 2 represents the core elements of an ongoing counseling process for patients with multimorbidity in primary care. Central to the process is the sharing of realistic treatment goals by physician and patient. They result from a thorough interaction assessment of conditions and treatments – a necessary starting point for both presenting and continuing problems – and a prioritization of health problems that takes into account patient preferences. Individualized management realizes the best (available) options of care (diagnostics and treatment, but also in primary and secondary prevention) to achieve the goals. Goal attainment is followed-up in accordance with a re-assessment during planned visits. The occurrence of new or changed conditions, such as an increase in severity, or a changed context of the patient may provoke the (re-)start of the process. The main forces driving the care of patients with multimorbidity are interacting conditions (and treatments), as opposed to patients with a single disease (even if complicated). The process is not necessarily sequential, as patient’s preferences may change over time, or the individualized management may have to be corrected, e.g., due to arising intolerable side effects.

#### Interaction assessment

In contrast to patients with single diseases, in patients with multimorbidity a broad variety of potential interactions between diseases and treatments may occur which may worsen the course of the disease(s), cause (avoidable) symptoms, and complicate diagnostic work-up as well as treatment and prevention [24]. Therefore, relevant mechanisms which have to be checked separately are drug-drug, drug-disease, and disease-disease interactions. Apart from possible adverse drug effects, which are more likely in multiple medications [25], complex medication regimens should trigger awareness of the increased risk of reduced adherence or under-treatment that are both typical risks of polypharmacy [26,27].

It is important to keep a list of all individual diagnoses and to assess their severity and impact on quality of life and functioning. Symptoms such as pain, fatigue, shortness of breath, or dizziness have a great impact on quality of life and life satisfaction and thus – likely – on patient preferences [28]. Medication that is currently being taken should be reviewed regularly [29], and along with the assessment of overall treatment burden, including pharmacological and non-pharmacological treatments, a list of other physicians and therapists involved in the patient’s care should be updated.

An active monitoring for signs and symptoms of psychological problems, mental disorders, and cognitive dysfunction is essential, as is the identification of social circumstances that may influence care seeking, patient health, and the need for assistance in activities of daily living [30,31]. In patients with multiple diseases, the balance between resources and burden may be disrupted by diseases, such as depression, anxiety, or by contextual circumstances (living conditions, level of social support, loneliness, or financial constraints) [1,9,32]. Health literacy is challenged when complex health regimens are put in place. Patients’ social participation, functional autonomy, coping strategies, and health care-seeking behavior should also be elicited and considered, as these provide valuable contextual information that may support clinical decision making and care planning.

#### Prioritization and patient’s preferences

If the interactions of planned treatment result in more harm than good, or treatment goals compete with one another, or the total treatment burden is inacceptable, then priorities must be set. Such health care decisions need to be made within the context of patients’ values and preferences. Patient’s preferences should be thoroughly elucidated, and priorities and realistic treatment goals should be agreed upon. Family physicians should be aware of their own potentially differing preferences [11] that may be the result of extrinsic factors, such as the fear of financial or legal threats.

The patient’s prognosis, in terms of physical and mental functioning, quality of life, and life expectancy, should always be taken into consideration [18]. Health outcomes shift from disease-specific to generic and patient’s values often swing from life expectancy to quality of life. Family physicians may assess preferences on the basis of a discussion and rank the outcomes accordingly [33]. Patients may prioritize desired outcomes, such as symptom relief, preservation of physical, mental, and social functioning, or disease prevention, but also the avoidance of adverse outcomes, such as nausea, drowsiness, dizziness, lethargy, or confusion [34]. Family physicians should enquire about these preferences but also assess the acceptance of several treatments and the ability of the patient to manage them [9,32]. Clarifying the patient’s preferences will require an understanding of their concerns – is it the symptoms or the potential consequences that trouble them most? Although, patients may want more (or less) responsibility for their health decision [35,36], a neglected preference can be harmful [37].

The treatment goals should ideally be defined in terms of time, that is, at what point in time this goal should be reached or a benefit obtained. Such clarifications may support monitoring and re-discussing priorities when goals are not attained or not at the expected time. In particular, in typically long-lasting family physician-patient relationships [19], preferences, priorities, and treatment goals have to be re-assessed regularly, as they may change, or even be reversed when, for example, new diseases develop or contextual changes occur [38].

#### Individualized management and follow-up

**Figure 3:**
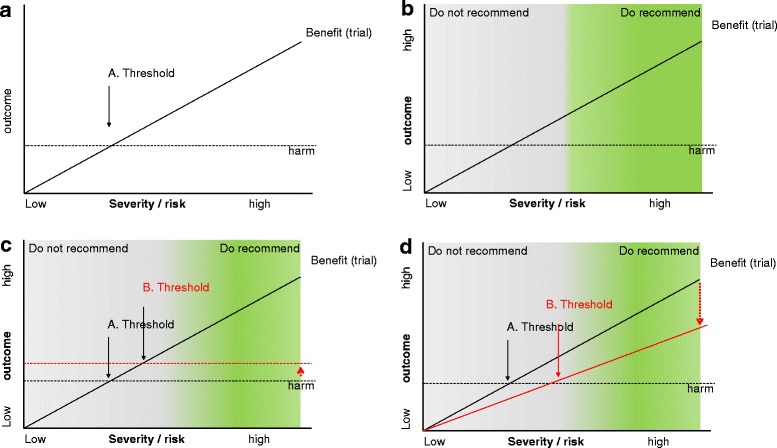
**A general model for treatment decisions. (a)** A net benefit only occurs when the individual patient’s risk or disease severity is sufficiently high to be to the right of the treatment threshold, where the benefit and harm lines cross. **(b)** In most cases, there is no clear cut-off between recommended and not recommended treatments. For example, for a patient with both rheumatoid arthritis and heart failure, any benefit of non-steroidal anti-inflammatory drugs needs to be weighed against the higher risk of fluid retention and its effects on heart failure [41]. **(c)** Some chronic diseases, in particular renal and liver failure, narrow the therapeutic window of many drugs and hence increase the likelihood of harm. **(d)** Chronic diseases can attenuate the relative benefit of treatment such as statin therapy in patients with chronic kidney disease receiving dialysis [42].

After the prioritization of problems, a care plan which sets out monitoring, treatment, prevention, and (self-)management advice should be developed to meet shared and realistic treatment goals. A central issue is whether the expected benefits of an intervention (diagnostic, therapeutic, or preventive) outweigh the likely downsides and harms to the individual patient. As a general rule, ‘single-disease’ patients with more severe diseases or at a higher risk of negative health outcomes, have a greater potential for benefit. Hence, benefits are more likely to outweigh harms; whereas low risk patients may expect less benefit but are exposed to the same potential harms [39]. Multimorbidity can complicate this simple model by modifying the patient’s risk, harms, or even the potential treatment benefits. The modifying factors can include both the chronic diseases themselves and their treatment (Figure 3). Furthermore, time to benefit should be considered, taking into account the patient’s preferences and expected survival [40].

The simple model may be complicated with multimorbidity by drug-drug, drug-disease, and disease-disease interactions, and further complicated by the paucity of data about their extent. The first consequence is that we should generally be more conservative when introducing additional treatments while at the same time remaining aware of the risk of under-treatment. Secondly, we have to anticipate unintended consequences of any new treatment that is to check for potential interactions *ex ante* and *ex post* (follow-up). The identification of interactions can be facilitated through collaboration with community pharmacists and the optimal use of technology. User-friendly applications, such as optimized support systems alerting for potentially inappropriate medication or interactions, are useful, but are incomplete without a judgment of their clinical relevance. Thirdly, complex medication regimens are challenging for patients to comply with. Sometimes simple solutions exist, such as altering preparations to modified release formulations or using simple prompts or reminders (e.g., dosette boxes) to assist patients.

The careful coordination of care – the often necessary involvement of different health care professionals at different levels and settings of care – is an important component of individualized care and should ensure continuity [19]. Appointments should be prioritized by applying a minimally disruptive approach [9] to meeting agreed treatment goals. Care plans for patients with multimorbidity are not static, but subject to continuous adaptation depending on changes in the prioritization of problems, goal attainment, or as a result of co-occurring events or altering contexts. It is also important that the patient has a family physician in charge of his overall health process [19]. Family physicians should be aware of new triggers (Figure 2), which should guide the focus of the following consultations.

#### Reflections on the case of Mr. P

At T2 (Figure 1), a beta-blocker was prescribed to Mr. P to slow down the progression of his coronary artery disease. This benefit outweighed potential harms of worsening his asthma. Mr. P agreed and his physician provided instructions for safety netting and regular follow-ups. At T3, a potential interaction between the beta-blocker and asthma was ruled out by lung function testing, and his cough was considered to be a symptom overlap caused by his deteriorating Parkinson’s disease. However, the ambulatory intensification of drug therapy led to new problems. Mr. P agreed to admission to specialized care. Although complicated by an adverse drug event due to a drug-disease interaction (amantadine and benign prostatic hyperplasia), his situation finally improved.

Over his life-course, treatment goals shifted from disease-specific (e.g., blood glucose) to generic (e.g., physical functioning). Mr. P still has a strong preference for survival (at good quality), and is willing and capable of coping with his diseases and treatment burden. In accordance with this and his general prognosis, prioritization has not led to a de-intensification of his treatment but to a critical selection of further preventive activities.

## Discussion

We have formulated a comprehensive longitudinal approach to the goal-oriented management of patients with multimorbidity in primary care, i.e., an approach addressing multiple consultations. For the heterogeneous group of patients with multimorbidity, there are no easy solutions that apply to all patients. Therefore, we have developed a set of principles that can be used to structure and enrich the approach to consultations as suggested by Stott and Davis and to improve patient outcomes using tailor-made approaches.

Clinicians and patients should realize that in a complex situation with multiple diseases and several treatments, there is no ‘single best’ choice of treatments. This may be the case with treatments which may have beneficial effects to one disease and the potential to cause harm in another at the same time. Although, physician and patient share the decision for this option, this does not necessarily prevent negative consequences. In other circumstances, patient and physician may share a decision against an effective therapy in order to reduce the treatment burden. This decision may well result in a preventable major event. Only little is known about patients and caregivers coping with the negative consequences of such actions [34,43].

Our principles are not intended to support a unidirectional de-intensification of treatment: a thorough assessment of presenting and continuing problems may identify under-treatment, or the need for intensification, and the elucidation of patient’s preferences may show that the patient does not perceive multiple drugs as an unbearable burden. Prioritization is a process of assigning priorities to problems or tasks but does not necessarily mean a reduction.

Furthermore, our principles support a critical approach to guidelines in a patient with multimorbidity, as we have currently little to replace them [44]. The application of guidelines may be safe and effective when potential interactions are checked for, shared treatment goals are met, and the effects are closely followed-up on. In patients with multimorbidity, the use of guidelines will have to be further considered and more selective to prevent clinical management from being “*inappropriately driven by algorithmic protocols*, *top-down directives and population targets*” [45]. This may also have consequences for guideline-based processes such as disease management programs and financial incentives in health care systems.

### Limitations and implications for further research

We do not pretend to have a final solution to the complex problem of managing patients with multimorbidity in primary care for several reasons. Firstly, although we received feedback from GPs and primary care researchers, the application of the proposed key principles has not yet been tested and the development process did not involve the patient. Implementation may also be difficult to achieve within the constraints of a 10 minute consultation, but it may be possible to integrate it into existing models of care (e.g., the Chronic Care Model [46,47]) and develop interventions within (pro-active) primary care teams and across health care providers which may be effective in multimorbidity management [48]. Secondly, multimorbidity in itself is not a homogeneous condition. Not only the number and severity of conditions, but also other factors such as social issues or mental illness may determine multimorbidity [49,50]. Thirdly, the implementation of these principles is rendered more complex by our current modest evidence-base and limited theory.

Given these limitations, the core elements of the Ariadne principles outlined in Figure 2 suggest several research priorities. The interaction assessment may be disappointing due to the shortcomings in the evidence about interactions and their clinical relevance. High-quality and integrating information technology systems could help, but further work is needed to optimize the benefit of this modality [51,52]. Furthermore, the gap of knowledge and development of proper theoretical models on the prioritization hamper this process. Although patient preferences are embraced in concepts such as patient-centeredness and goal-oriented care [53], little is known about how to elicit (and construct) patient’s health-related preferences when multiple trade-offs complicate the decision and on how patients cope with negative consequences. In addition, evidence is sparse on the methods and impact of individualized management. Outcomes studied are often disease-specific and less meaningful for decision making in patients with multiple diseases [54].

Our principles may encourage physicians to actively consider multimorbidity when making medical decisions. However, the principles need further critical reviewing, followed by empirical testing using case vignettes, case conferences, role plays, and directly observed consultations, involving GPs and patients. In addition, further work on prioritization is necessary to gain a better understanding of determinants and decision-making processes, and to provide appropriate tools supporting interaction assessment and a communication process that results in physician and patient sharing realistic treatment goals.

## Conclusions

We have developed the Ariadne principles to be adopted by family physicians in daily practice. These principles may also be incorporated into educational programs on the care of patients with multimorbidity in both medical education and vocational training. The further refinement and elaboration of these principles should be based on experiences gained from their practical application.

## Consent

Written informed consent was obtained from the patient for publication of this Case report and any accompanying images. A copy of the written consent is available for review by the Editor of this journal.

## Box 1: The Ariadne principles – practical hints

### Assess potential interactions – the patient’s conditions and treatments, constitution and context

- Keep a list of all current conditions, assess their severity and impact, and review the medication currently taken.
- Actively monitor for signs of anxiety, distress and depression, or cognitive dysfunction, including problems of addiction and non-specific signs or symptoms such as sleeping problems, loss of appetite, or hydration problems.
- Elicit and consider social circumstances, financial constraints, living conditions and social support, health literacy, functional autonomy, and coping strategies.
- List other physicians and therapists involved in the patient’s care and assess overall treatment burden.

### Elicit preferences and priorities – the patient’s most and least desired outcomes

- Elicit preferences for generic health outcomes, such as survival, independence, pain, and symptom relief including palliative care needs, and be aware of your own (implicit) preferences, as they may not be the same as the patient’s.
- If applicable, consider preferences of informal caregivers or family.
- Agree on a realistic treatment goal with the patient (and patient’s caregiver if appropriate).

### Individualize management to reach the negotiated treatment goals

- Weigh up whether the expected benefits of treatment (and prevention) outweigh the likely downsides and harms, given the individual patient’s risk level and preferences.
- Assess the incremental and combined treatment burden of the patient (and caregiver, if applicable).
- Consider self-management according to the patient’s needs and capabilities.
- Provide instructions for safety-netting such as symptoms of side effects and recommendations about the appropriate management.
- Agree with the patient on the schedule for follow-up visits to evaluate goal attainment and re-assess interactions.
- Consult other health care providers and informal caregivers who are involved with the patient. Ideally, all health care providers involved are informed about treatment decisions or have access to information.

## Additional file

Additional file 1:
**Web-based supplement.**
