# Supplementary material for: The Ariadne principles: how to handle multimorbidity in primary care consultations
Source: BMC Med. 2014 Dec 8;12:223. doi: 10.1186/s12916-014-0223-1 (PMC4259090; doi:10.1186/s12916-014-0223-1)
Supplement: Additional file 1: — Web-based supplement. [file 12916_2014_223_MOESM1_ESM.docx]

## Web-based Supplement

Results of the written feedback process:

| **Participants**  Males:  Australia:  Canada:  Germany:  Netherlands:  Spain:  United Kingdom: | N=24  14 (58%)  4 (16%)  5 (21%)  7 (29%)  3 (12.5%)  2 (8%)  3 (12.5%) |
| --- | --- |
| **Appropriateness** (mv=2)  Principles are appropriate | 20 (91%) |
| **Usability** (mv=2)  Principles are helpful: Median (Interquartilrange, IQR) | 5 (4,5) |
| **Comprehensiveness**  Nothing to delete (mv=3)  Nothing to add (mv=4) | 19 (90%)  13 (65%) |

Feedback was provided on (1) appropriateness (yes/no), (2) usability (from “6” – very helpful to “1” – not helpful at all), and (3) comprehensiveness (something/nothing to be deleted, something/nothing to be added) in ratings and free text comments.
